# An integrated skin cancer education program in renal transplant recipients and patients with glomerular disease

**DOI:** 10.1186/s12882-022-02997-z

**Published:** 2022-11-10

**Authors:** Zaw Thet, Alfred King-yin Lam, Shu-Kay Ng, Soe Yu Aung, Thin Han, Dwarakanathan Ranganathan, Stephanie Newsham, Jennifer Borg, Christine Pepito, Tien K. Khoo

**Affiliations:** 1 School of Medicine and Dentistry, Menzies Health Institute Queensland, Griffith University, Gold Coast, Queensland 4222 Australia; 2 Department of Nephrology, Central Queensland Hospital and Health Service, Emerald, Queensland Australia; 3 Faculty of Medicine, University of Queensland, Brisbane, Queensland Australia; 4 Pathology Queensland, Gold Coast University Hospital, Gold Coast, Queensland Australia; 5 Department of Oncology, Central Queensland Hospital and Health Service, Rockhampton, Queensland Australia; 6 Department of Nephrology, Metro North Hospital and Health Service, Herston, Queensland Australia; 7 School of Medicine, University of Wollongong, Wollongong, New South Wales Australia

**Keywords:** Education, Skin cancer, Glomerular disease, Renal transplant

## Abstract

Sun-protective strategies focusing on skin cancer awareness are needed in immunosuppressed patients at risk of skin cancers. The study aims to determine the effect of an integrated skin cancer education program on skin cancer awareness and sun-protective behaviours in renal transplant recipients (RTRs) and patients with glomerular disease (GD) treated with long-term immunosuppressants. A pilot prospective cohort study in Central Queensland, Australia was undertaken among adult RTRs and patients with GD, who completed survey questionaries on skin cancer and sun-health knowledge (SCSK), sun-protection practices and skin examination pre- and post-education. Fifty patients (25 RTRs, 25 patients with GD) participated in the study. All of them completed questionnaires at pre-, 3-month post-education and 92%(*n* = 46) at 6-month post-education. There was a significant increase in SCSK scores from baseline at 3-months (*p* < 0.001) and 6-months post-intervention (*p* < 0.01). Improved knowledge was retained for 6 months after education. There were changes in 2 of 8 photoprotective behaviours at 6 months. Interventional education enhanced regular self-skin examination rate (*p* < 0.001) as well as the frequency of full skin checks by general practitioners (GPs) (*p* < 0.001). Overall, RTRs had better compliance with sun-protective methods and higher skin examination rates by themselves and/ or GPs before and after the intervention of education compared to patients with GD. To conclude, an integrated skin cancer education program improved knowledge of skin cancer and skin health as well as the frequency of self-skin examination and formal skin assessments. However, improvement in patient compliance did not extend to other sun-protective practices.

**Supplementary Information:**

The online version contains supplementary material available at 10.1186/s12882-022-02997-z.

## Background

Ultraviolet radiation (UVR), a non-ionizing radiation emitted by the sun has been reported to be a major carcinogen responsible for most of the skin cancer because it damages the deoxyribonucleic acid (DNA) and causes genetic mutations [1]. The administration of immunosuppressive drugs increases the risk of UVR-related skin cancers in patients with glomerular diseases (GD) [2–4] and renal transplant recipients (RTRs) [5, 6]. Among kidney diseases, glomerular diseases (nephrotic syndrome and glomerulonephritis) and renal transplantation are two main conditions that require long-term immunosuppressants. Although data on skin cancer and associated risk factors in the transplant setting is well established, there is limited data to determine the risk of skin cancer in patients with GD treated with immunosuppressants [2–4].

Incidence of SCC in solid organ transplant recipients was 50–250 times higher whereas that of BCC 10 times higher when compared to the general population [5]. Risk factors for skin cancers are long duration and high intensity of immunosuppression, ultraviolet sun exposure, previous history of skin cancer, fair skin complexion or phototype, age at transplantation, smoking, male sex, and viral infection with human papillomavirus (HPV). Amongst these risk factors, a modifiable one in the prevention of skin cancer in transplant recipients is the reduction of exposure to UVR.

To date, skin cancer awareness and sun-protective behaviours of patients with GD are almost unknown. Many studies on skin cancer knowledge, sun-protective behaviours and practices in organ transplant recipients revealed suboptimal findings [7–29]. Previous studies published in Australia did not include regional transplant recipients who often have lower education attainment and employment opportunities which may affect an individual’s awareness of skin cancers and sun-protective measures [8, 23]. Studies have also suggested that patient compliance with sun-protective measures may increase if education is emphasized repeatedly after immunosuppressant exposure [8–11, 13–16, 20]. This is particularly important in high-risk patients with light-skinned (Fitzpatrick skin type I-II) and a history of chronic ultraviolet radiation exposure, skin cancers and higher immunosuppressant exposure.

The aim of this prospective cohort study is to assess skin cancer and sun health knowledge, sun-protective practices during outdoor activities and regular skin examination by themselves or health practitioners among immunocompromised RTRs and patients with GD before and after an integrated skin cancer educational program.

## Patients and methods

This pilot study with a quasi-experimental design involved RTRs and patients with GD who attended renal clinics in Central Queensland Hospital and Health Service (CQHHS) in Australia from 3 November 2020 to 30 April 2021. Informed consent was obtained from all subjects and/or their legal guardian(s). Follow-up was conducted until the end of the study period on 31 December 2021. Participants were consecutive adult (≥18 years) RTRs and patients with GD treated with immunosuppressants and under the immediate care of the Rockhampton Renal Unit in Queensland, Australia. This study was undertaken in accordance with the national statement on ethical conduct in human research and Declaration of Helsinki. It was approved by the National Ethics Committee and the local governance authority (HREC/2020/QCQ68183) in Central Queensland Hospital and Health Service.

Only RTRs were included in this study as heart and lung transplant recipients are not followed-up by the Central Queensland Renal Unit. All adult RTRs with first-time kidney graft whose transplant surgery performed in Australia and post-transplant care occurred under CQHHS were included in the study. Patients with second or subsequent renal transplantation and those who lacked sufficient capacity or had significant cognitive impairment were excluded from the study. The GD group comprised of adult patients with renal biopsy proven glomerular disease treated with long-term immunosuppressants. Exclusion criteria in the GD cohort were those with significant cognitive impairment/ dementia, glomerular disease due to infection, paraproteinemia, multiple myeloma, light disease, amyloidosis or paraneoplastic glomerulopathy as infections especially HBV, HCV and HIV, haematological malignancies, and solid cancers and associated treatments can affect participation of patients at pre- and post-intervention.

**Fig. 1:**
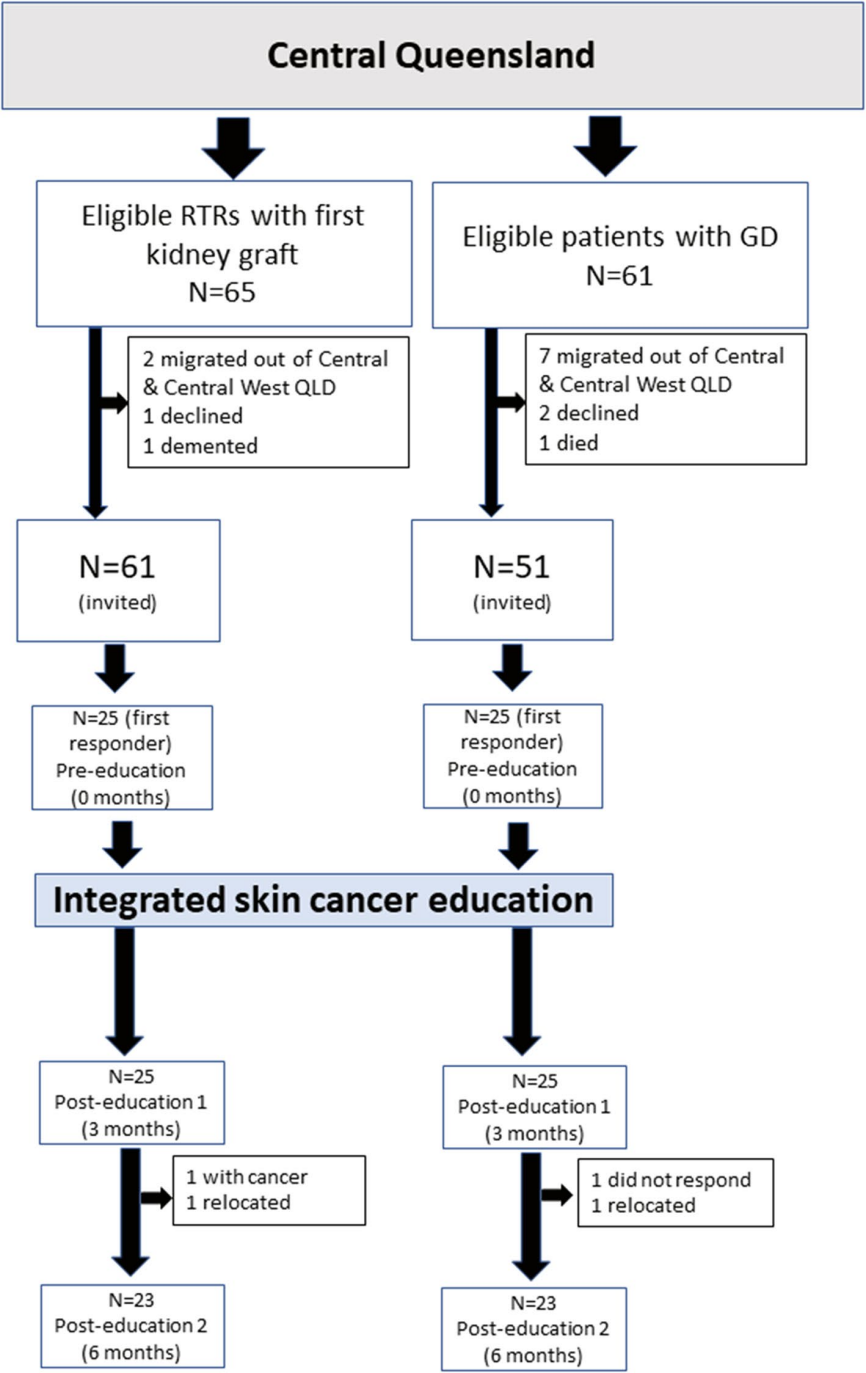
Participant recruitment. RTRs: renal transplant recipients; GD: glomerular diseases

All RTRs and patients with GD in CQHHS who met inclusion criteria were approached to participate in the pilot study. Figure 1 details participant recruitment. Of the eligible 116 patients (65 RTRs and 51 patients with GD), 25 patients from each cohort who consented first were recruited in an integrated skin cancer education program. A pilot program was developed in Central Queensland after a reviewing information available from Cancer Council Australia (www.cancer.org.au), Cancer Council Victoria (www.sunsmart.com.au), Cancer Research UK (www.cancerresearchuk.org), American skin cancer foundation (www.skincancer.org), literature that emphasized improving individual awareness on skin cancer risk could be achieved by multiple educational methods including a video, individualized education about self-skin examinations, sun protection practices, and the importance of seeing a health practitioner for skin checks [7–29]. The program comprised of the following:

- A booklet from the Skin Cancer Foundation to understand skin cancer
- A video and a brochure from the Skin Cancer Foundation describing how to perform a self-skin examination.
- One to one systematic self-skin examination instruction with a transplant coordinator or researcher.
- Patient educational brochure on optimal sun protection practices.
- One to one appointment with a researcher or renal pharmacist with regards to immunosuppressant-related cancer risks.
- A diary on skin health in which patients and health practitioners can document skin examination findings, and skin cancer reports.

All participants were asked to complete a questionnaire on Skin Cancer and Sun-health Knowledge (SCSK) scale and sun-protective practices prior to the intervention (pre-education), at 3-months (post-education 1) and 6-months (post-education 2) following the intervention. The SCSK scale, a validated tool assesses skin cancer and sun health knowledge and encompasses five broad domains of knowledge: sun protection, tanning, skin cancer risk factors, prevalence of skin cancer, and signs of skin cancer. The SCSK scale includes 15 true-false items and 10 multiple choice items, with a possible score range of 0 to 25 with higher score indicating higher knowledge. A questionnaire assessing the use of sun-protective practices consists of an eight-item tool, with “yes” or “no” using photoprotective measures in outdoor activities, which generates a possible score range of 0 to 8; higher score indicates better sun protection. Participants who used sunscreen were asked to specify their frequency of sunscreen use and seasons during which they were used.

Continuous variables were reported as means with standard deviations, or medians and interquartile ranges, whereas categorical variables were expressed as number (values) and the percentage. Categorical variables were compared between groups using *Chi*-square test or Fisher’s exact test. The independent samples *t-*test was used to compare the means between groups on continuous variables. A paired samples *t*-test was used to compare the means of quantitative data before and after education for the same subjects. McNemar’s test and McNemar-Bowker test of symmetry (when there are more than two categories) were used on paired nominal data for the same subjects. Level of significance was set at a two-tailed *p* value of ≤0.05. All analyses were performed using Statistical Package for the Social Sciences (SPSS version 28, IBM, New York, USA).

## Results

(A) Survey response

A total number of 50 patients (25 RTRs, 25 patients with GD, *n* = 100%) completed a questionnaire at pre-education and 3-month post-education. Of these, 46 (23 RTRs and 23 patients with GD, *n* = 92%) completed a questionnaire at 6-month post-education.

(B) Demographics

The median age of all participants was 62 years (standard deviation, SD = 13.5 years) and 58% were male. 94% of all participants were Caucasians and 54% (26 Caucasians and 1 Asian) had a history of skin cancer. The mean time spent in formal education was 11.9 years (SD = 3.3 years). The median time since the first dose of immunosuppressants was 62 months (interquartile range, IQR = 36.8–108.5 months). There was no significant difference in mean age, sex, race, time spent in formal education and median time since the first dose of immunosuppressants between RTR cohort and GD group. 72% of RTRs and 36% of patients with GD had a history of skin cancer (*p* = 0.022). Participant demographics are shown in Table 1.

(III) Skin cancer and sun health knowledge

**Table 1:** Demographics of participants

	All participants (***N*** = 50)	RTRs (***N*** = 25)	GD Patients (***N*** = 25)	***p***-value
Median age (Min-Max) (SD), years	62 (20–78)	61 (37–74)	65 (20–78)	0.253
	(13.5)	(11.38)	(15.3)	
Sex (N)				
Female	21 (42.0)	9 (36.0)	12 (48.0)	0.567
Male	29 (58.0)	16 (64.0)	13 (52.0)	
Race (N, %)				
Caucasian	47 (94.0)	50 (100.0)	22 (88.0)	0.235
Asian	3 (6.0)	0	3 (12.0)	
Personal history of skin cancer (N, %)				
Yes	27 (54.0)	18 (72.0)	9 (36.0)	**0.022**
No	23 (46.0)	7 (28.0)	16 (64.0)	
Formal education (Min-Max) (SD), years	11.9 (4.0–19.0) (3.3)	11.9 (4.0–19.0) (3.4)	11.9 (6.0–18.5) (3.3)	0.983
Median time since the first dose of immunosuppressants (IQR), months	62.0 (36.8–108.5)	63.0 (35.5–111.5)	61.0 (38.5–97.5)	0.512

*RTR* Renal transplant recipients

*GD* Glomerular diseases

*Min* Minimum

*Max* Maximum

*SD* Standard deviation

*IQR* Interquartile range

The mean SCSK score of all participants at baseline was 19.3 (SD = 3.3). The SCSK score was improved at 3 months (all participants 21.0, *p* < 0.001, RTR cohort 21.8, *p* = 0.004 and GD group 20.2, *p* = 0.039) and 6 months (all participants 20.9, *p* < 0.001, RTR cohort 21.0, *p =* 0.040 and GD group 20.9, *p* = 0.004) after the education. SCSK scores are detailed in Table 2. There was no significant difference in SCSK score between RTRs and patients with GD before receiving skin cancer education (mean: 19.7 vs 19.0, *p* = 0.473) and 6 months after education (mean score 21.0 vs 20.9, *p* = 0.939) as shown in Table 3. However, RTRs had higher mean SCSK score at 3 months post-education, compared with patients with GD (21.8 vs 20.2, *p* = 0.031). However, this result was not significant (*p* = 0.278) when accounting for SCSK scores pre-education (change in SCSK score from pre-education at 3 months: mean = 2.2 (RTRs) vs mean = 1.2 (GD); see Table 3).

(IV) Outdoor activities and photoprotective behaviours

**Table 2:** Skin Cancer and Sun health Knowledge of participants

**A: All participants**				
**Intervention**	**N**	**Mean SCSK score (SD)**	***p*** **value**	**Change in SCSK score from baseline (95%CI)**
Pre-education (0 months)	50	19.3 (3.3)	Ref	Ref
Post-education 1 (3 months)	50	21.0 (2.7)	**< 0.001**	1.9 (0.8–2.6)
Post-education 2 (6 months)	46	20.9 (1.8)	**< 0.001**	1.6 (0.8–2.5)
**B: RTR cohort**				
**Intervention**	**N**	**Mean SCSK score (SD)**		**Change in SCSK score from baseline (95%CI)**
Pre-education (0 months)	25	19.7 (3.1)	Ref	Ref
Post-education 1 (3 months)	25	21.8 (1.5)	**0.004**	2.2 (0.8–3.6)
Post-education 2 (6 months)	23	21.0 (1.6)	**0.040**	1.3 (0.1–2.5)
**C: GD cohort**				
**Intervention**	**N**	**Mean SCSK score (SD)**		**Change in SCSK score from baseline (95%CI)**
Pre-education (0 months)	25	19.0 (3.5)	Ref	Ref
Post-education 1 (3 months)	25	20.2 (3.4)	**0.039**	1.2 (0.1–2.3)
Post-education 2 (6 months)	23	20.9 (1.9)	**0.004**	2.0 (0.7–3.3)

*SCSK* Skin cancer and sun health knowledge

*SD* Standard deviation

*95%CI* 95% confident interval

*RTR* Renal transplant recipients

*GD* Glomerular diseases

**Table 3:** Comparison of skin cancer awareness and sun-protection (RTRs vs GD patients)

**Skin cancer awareness**			
	**RTRs** **(*N*** **= 25)**	**GD Patients** **(*N*** **= 25)**	***p-*value**
**Mean SCSK score** (SD)			
Pre-education	19.7 (3.1)	19.0 (3.5)	0.473
Post-education (3-months)	21.8 (1.5)	20.2 (3.4)	**0.031**
Post-education (6-months)	21.0 (1.6)	20.9 (1.9)	0.939
**Change in SCSK score from baseline** (95%CI)			
Pre-education	Ref	Ref	
Post-education (3-months)	2.2 (0.8–3.6)	1.2 (0.1–2.3)	0.278
Post-education (6-months)	1.3 (0.1–2.5)	2.0 (0.7–3.3)	0.391
**Sun Protection**			
	**RTRs** **(*N*** **= 25)**	**GD Patients** **(*N*** **= 25)**	***p-*value**
**Mean total number of sun-protective methods used** (SD)			
Pre-test	5.1 (2.3)	4.4 (2.0)	0.239
Post-education (3-months)	5.4 (2.1)	4.0 (2.3)	**0.023**
Post-education (6-months)	6.0 (1.9)	4.2 (2.2)	**0.004**
**Change in total number of sun-protective methods used from baseline** (95%CI)			
Pre-test	Ref	Ref	
Post-education (3-months)	0.3 (−0.4, + 1.0)	−0.4 (−1.2, + 0.4)	0.082
Post-education (6-months)	1.0 (0.1–1.9)	−0.1 (−1.0, + 0.8)	**0.047**

*RTRs* Renal transplant recipients

*GD* Glomerular diseases

*SCSK* Skin cancer and sun health knowledge

*SD* Standard deviation

*95%CI* 95% confident interval

**Fig. 2:**
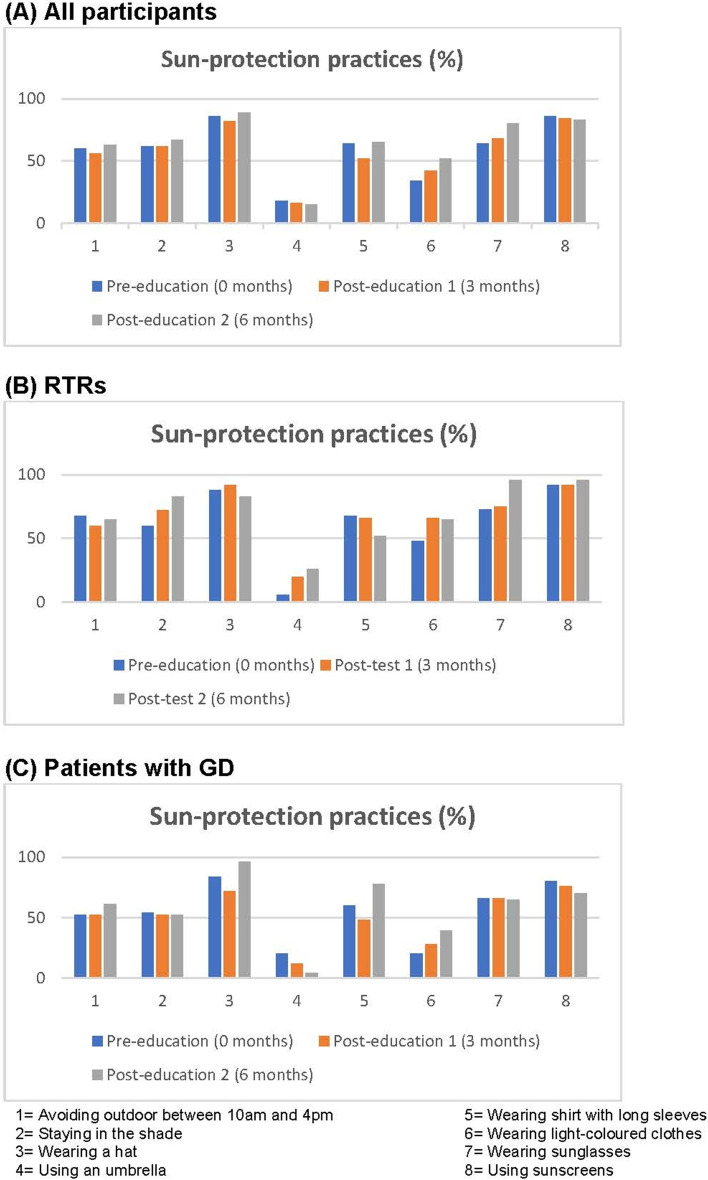
Sun-protective practices by groups

**Fig. 3:**
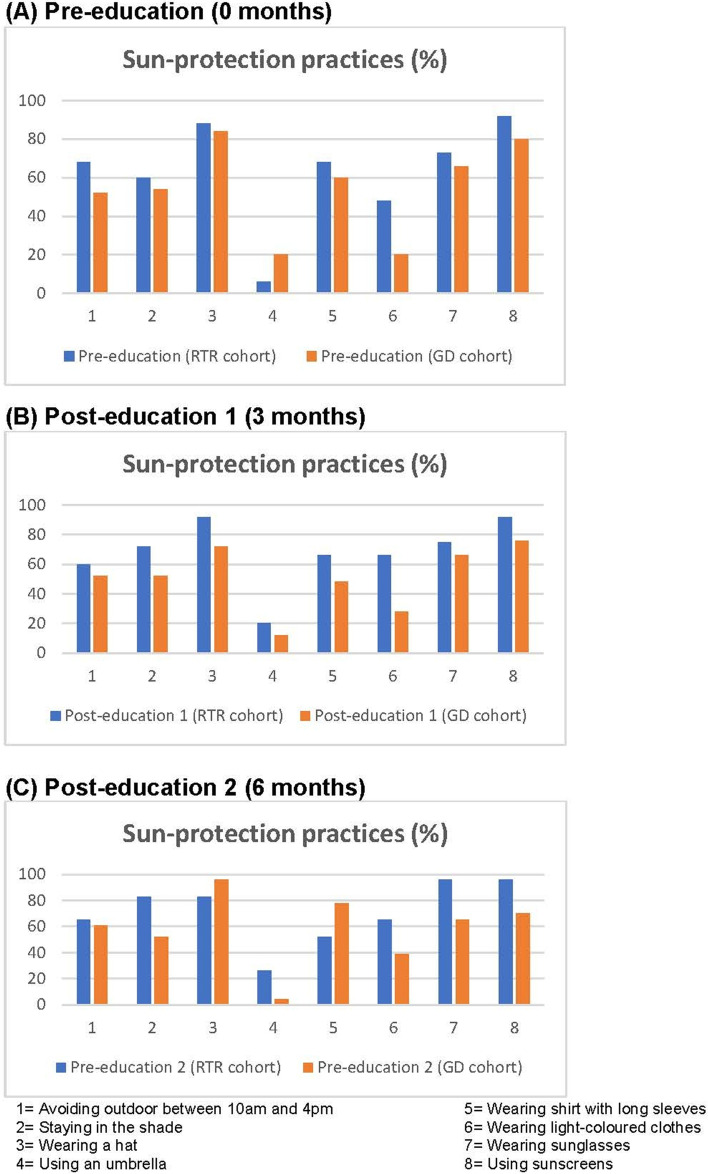
Sun-protective practices (RTRs versus patients with GD)

The frequency of participant outdoor activities for work or recreation or exercise under the sun and compliance with sun-protective practices used by the patients are summarized in Table 4 and Figs. 2 to 3. The frequency of outdoor activities of all participants did not change before and after education. In the whole participant cohort and GD cohort, the mean total number of sun-protective methods used also did not changed between baseline and 3-months (whole cohort, 4.8 vs 4.7, *p* = 0.757, GD cohort, 4.4 vs 4.0, *p* = 0.268) and 6-months post-intervention (whole cohort, 4.8 vs 5.1, *p* = 0.170, GD cohort, 4.4 vs 4.2, *p* = 0.842) (Table 4). However, the number of sun-protective methods practised did increase at 6 months post-education in the RTR cohort (5.1 vs 6.0, *p* = 0.038). There was no difference in mean total number of sun-protective methods used between RTR cohort and GD cohort at pre-education (5.1 vs 4.4, *p* = 0.239) whereas there was a difference demonstrated at 3 months (5.4 vs 4.0, *p* = 0.023) and 6 months (6.0 vs 4.2, *p* = 0.004) post-education (Table 3). Relative to the number of sun-protective methods practised pre-education, the mean number of sun-protective methods used changed by 0.3 (RTRs) and − 0.4 (GD) at 3 months (*p* = 0.082), and at 6 months by 1.0 (RTRs) and − 0.1 (GD) with *p* = 0.047. Overall, RTRs had better compliance with sun-protective practices before and after the education when compared to patients with GD as shown in Table 4 and Figs. 2 to 3. In the whole cohort, a higher number of participants wore light coloured clothes (*p* = 0.039) and sunglasses (*p* = 0.039) at 6 months after education, compared to pre-education. In the RTR cohort, the number of patients who wore sunglasses (*p* = 0.031) and stayed in the shade (*p* = 0.031) increased. In GD cohort, there was no change in compliance with each sun-protective practice after the education.

(E) Skin examination

**Table 4:** Outdoor activities and compliance with sun-protective practices

	All participants	***p*** value	RTR cohort	***p*** value	GD Cohort	***p*** value
**Outdoor activity under the sun** (N, %)						
Pre-education (0 months)		Ref		Ref		Ref
Daily/Almost daily	32/50 (64%)		16/25 (64%)		16/25 (64%)	
Weekly	7/50 (14%)		5/25 (20%)		2/25 (8%)	
Fortnightly	4/50 (8%)		3/25 (12%)		1/25 (4%)	
Monthly	1/50 (2%)		0/25 (0%)		1/25 (4%)	
Never/Almost Never	6/50 (12%)		1/25 (4%)		5/25 (20%)	
Post-education 1 (3 months)		0.340		n/a		0.261
Daily/Almost daily	28/50 (56%)		14/25 (56%)		14/25 (56%)	
Weekly	8/50 (16%)		5/25 (20%)		3/25 (12%)	
Fortnightly	5/50 (10%)		3/25 (12%)		2/25 (8%)	
Monthly	2/50 (4%)		1/25 (4%)		1/25 (4%)	
Never/Almost Never	7/50 (14%)		2/25 (8%)		5/25 (20%)	
Post-education 2 (6 months)		0.402		n/a		0.549
Daily/Almost daily	25/46 (54%)		13/23 (57%)		12/23 (52%)	
Weekly	7/46 (15%)		4/23 (17%)		323 (13%)	
Fortnightly	3/46 (7%)		2/23 (9%)		1/23 (4%)	
Monthly	6/46 (13%)		3/23 (13%)		3/23 (13%)	
Never/Almost Never	5/43 (11%)		1/23 (4%)		4/23 (18%)	
**Mean total number of sun-protective methods used** (N, SD)						
Pre-education	4.8 (SD 2.1)	Ref	5.1 (2.3)	Ref	4.4 (2.0)	Ref
Post-education 1	4.7 (SD 2.3)	0.757	5.4 (2.1)	0.403	4.0 (2.3)	0.268
Posy-education 2	5.1 (SD 2.2)	0.170	6.0 (1.9)	**0.038**	4.2 (2.2)	0.842
**Sun-protective methods** (N, %)						
Avoiding outdoor between 10 am and 4 pm						
Pre-education						
No	20/50 (40%)	Ref	8/25 (32%)	Ref	12/25 (48%)	Ref
Yes	30/50 (60%)		17/25 (68%)		13/25 (52%)	
Post-education 1						
No	22/50 (44%)	0.687	10/25 (40%)	0.687	12/25 (48%)	1.000
Yes	28/50 (56%)		15/25 (60%)		13/25 (52%)	
Post-education 2						
No	17/46 (37%)	1.000	8/23 (35%)	1.000	9/23 (39%)	0.687
Yes	29/46 (63%)		15/23 (65%)		14/23 (61%)	
Staying in the shade						
Pre-education						
No	19/50 (38%)	Ref	10/25 (40%)	Ref	9/25 (36%)	Ref
Yes	31/50 (62%)		15/25 (60%)		16/25 (54%)	
Post-education 1						
No	19/50 (38%)	1.000	7/25 (28%)	0.453	12/25 (48%)	0.508
Yes	31/50 (62%)		18/25 (72%)		13/25 (52%)	
Post-education 2						
No	15/46 (33%)	0.581	4/23 (17%)	**0.031**	11/23 (48%)	0.453
Yes	31/46 (67%)		19/23 (83%)		12/23 (52%)	
Wearing a hat						
Pre-education						
No	7/50 (14%)	Ref	3/25 (12%)	Ref	4/25 (16%)	Ref
Yes	43/50 (86%)		22/25 (88%)		21/25 (84%)	
Post-education 1						
No	9/50 (18%)	0.687	2/25 (8%)	1.000	7/25 (28%)	0.375
Yes	41/50 (82%)		23/25 (92%)		18/25 (72%)	
Post-education 2						
No	5/46 (11%)	0.625	4/23 (17%)	0.500	1/23 (4%)	1.000
Yes	41/46 (89%)		19/23 (83%)		22/23 (96%)	
Using an umbrella						
Pre-education						
No	41/50 (82%)	Ref	21/25 (84%)	Ref	20/25 (80%)	Ref
Yes	9/50 (18%)		4/25 (6%)		5/25 (20%)	
Post-education 1						
No	42/50 (84%)	1.000	20/25 (80%)	1.000	22/25 (88%)	0.625
Yes	8/50 (16%)		5/25 (20%)		3/25 (12%)	
Post-education 2						
No	39/46 (85%)	1.000	17/23 (74%)	0.687	22/23 (96%)	0.375
Yes	7/46 (15%)		6/23 (26%)		1/23 (4%)	
Wearing shirts with long sleeves						
Pre-education						
No	18/50 (36%)	Ref	8/25 (32%)	Ref	10/25 (40%)	Ref
Yes	32/50 (64%)		17/25 (68%)		15/25 (60%)	
Post-education 1						
No	24/50 (48%)	0.146	11/25 (44%)	0.375	13/25 (52%)	0.453
Yes	26/50 (52%)		14/25 (66%)		12/25 (48%)	
Post-education 2						
No	16/46 (35%)	1.000	11/23 (48%)	0.625	5/23 (22%)	0.687
Yes	30//46 (65%)		12/23 (52%)		18/23 (78%)	
Wearing light coloured clothes						
Pre-education						
No	33/50 (66%)	Ref	13/25 (52%)	Ref	20/25 (80%)	Ref
Yes	17/50 (34%)		12/25 (48%)		5/25 (20%)	
Post-education 1						
No	29/50 (58%)	0.289	11/25 (44%)	0/625	18/25 (72%)	0.625
Yes	21/50 (42%)		14/25 (66%)		7/25 (28%)	
Post-education 2						
No	22/46 (48%)	**0.039**	8/23 (35%)	0.453	14/23 (61%)	0.063
Yes	24/46 (52%)		15/23 (65%)		9/23 (39%)	
Wearing sunglasses						
Pre-education						
No	18/30 (36%)	Ref	7/25 (27%)	Ref	11/25 (44%)	Ref
Yes	32/50 (64%)		18/25 (73%)		14/25 (66%)	
Post-education 1						
No	16/30 (32%)	0.754	5/25 (25%)	0.625	11/25 (44%)	1.000
Yes	34/50 (68%)		20/25 (75%)		14/25 (66%)	
Post-education 2						
No	9/46 (20%)	**0.039**	1/23 (4%)	**0.031**	8/23 (35%)	0.687
Yes	37/46 (80%)		22/23 (96%)		15/23 (65%)	
Using sunscreens						
Pre-education						
No	7 (14%)	Ref	2/25 (8%)	Ref	5/25 (20%)	Ref
Yes	43 (86%)		23/25 (92%)		20/25 (80%)	
Post-education 1						
No	8 (16%)	1.000	2/25 (8%)	1.000	6/25 (24%)	1.000
Yes	42 (84%)		23/25 (92%)		19/25 (76%)	
Post-education 2						
No	8/46 (17%)	1.000	1/23 (4%)	1.000	7/23 (30%)	0.687
Yes	38/43 (83%)		22/23 (96%)		16/23 (70%)	
Mean total number of seasons by sunscreen use (N, SD)						
Pre-education	2.6 (SD 1.6)	Ref	3.1 (1.4)	Ref	2.1 (1.7)	Ref
Post-education 1	2.8 (SD 1.6)	0.322	3.4 (1.3)	0.335	2.2 (1.6)	0.660
Post-education 2	2.7 (SD 1.7)	0.437	3.5 (1.2)	0.153	1.9 (1.7)	0.601
Number of seasons by sunscreen use (N, %)						
Pre-education		Ref		Ref		Ref
4	25/50 (50%)		16/25 (64%)		9/25 (36%)	
3	4/50 (8%)				2/25 (8%)	
2	4/50 (8%)		2/25 (8%)		2/25 (8%)	
1	9/50 (18%)		2/25 (8%)		6/25 (24%)	
0	8/50 (16%)		3/25 (12%)		6/25 (24%)	
Post-education 1		0.322	2/25 (8%)	0.335		0.660
4	29/50 (58%)				9/25 (36%)	
3	4/50 (8%)		20/25 (80%)		3/25 (12%)	
2	3/50 (6%)		1/25 (4%)		3/25 (12%)	
1	7/50 (14%)		0/25 (0%)		5/25 (20%)	
0	7/50 (14%)		2/25 (8%)		5/25 (20%)	
Post-education 2		0.437	2/25 (8%)	0.153		0.601
4	27/46 (59%)		19/23 (84%)		8/23 (35%)	
3	1/46 (2%)		1/23 (4%)		0/23 (0%)	
2	3/46 (7%)		0/23 (0%)		3/23 (13%)	
1	7/46 (15%)		2/20 (8%)		5/23 (22%)	
0	8/46 (17%)		1/21 (4%)		7/23 (30%)	
Frequency of sunscreen use among users (N, %)						
Pre-education		Ref		Ref		Ref
Often/Always	18/50 (36%)		13/25 (52%)		5/25 (20%)	
Sometimes	20/50 (40%)		10/25 (40%)		10/25 (40%)	
Rarely	4/50 (8%)		0/25 (0%)		4/25 (16%)	
Never/Almost Never	8/50 (16%)		2/25 (8%)		6/25 (24%)	
Post-education 1		0.639		n/a		0.406
Often/Always	16/50 (32%)		13/25 (52%)		3/25 (12%)	
Sometimes	20/50 (40%)		8/25 (32%)		12/25 (48%)	
Rarely	6/50 (12%)		1/25 (4%)		5/25 (20%)	
Never/Almost Never	8/50 (16%)		3/25 (12%)		5/25 (20%)	
Post-education 2		0.745		n/a		0.406
Often/Always	20/46 (43%)		13/23 (56%)		7/23 (31%)	
Sometimes	16/46 (35%)		7/23 (31%)		9/23 (39%)	
Rarely	3/46 (7%)		2/23 (9%)		1/23 (4%)	
Never/Almost Never	7/43 (15%)		1/23 (4%)		6/23 (26%)	

n/a – the *p*-value cannot be computed due to the presence of zero observed value at pre-education

*RTR* Renal transplant recipients

*GD* Glomerular diseases

Regular self-skin examination rate was increased at 3 months (72%, *p* < 0.001) and 6 months (76%, *p* < 0.001) post-education in all participants when compared with the baseline rate at 28%. The same findings were noted in both, RTR (*p* = 0.004 at 3 months, *p* = 0.002 at 6 months), and GD (*p* < 0.001 at 3 and 6 months) cohorts. In addition, regular full skin checks by general practitioners (GPs) increased in the whole cohort from 44% at baseline to 74% at 3 months (*p* < 0.001) and 78% at 6 months (*p* < 0.001). This finding was also observed in the GD cohort (*p* < 0.001 at 3 and 6 months) after the education. In the RTR cohort, the rate of full skin checks by GPs at baseline was high at 80% and there was not much room for improvement at 3- and 6-months post-education. Although the rate at baseline increased to 92% at 3 months (*p* = 0.250) and 96% at 6 months *(p* = 0.219), these changes were not significant. Further details are shown in Table 5.

**Table 5:** Regular skin examination by patients and general practitioners

		All participants		RTR cohort		GD cohort	
Intervention	Type of skin examination	N (%)	***p*** value	N (%)	***p*** value	N (%)	***p*** value
Pre- education	**Self-examination**		Ref		Ref		Ref
	No	36/50 (72%)		12/25 (48%)		24/25 (96%)	
	Yes	14/50 (28%)		13/25 (52%)		1/25 (4%)	
Post-education 1			**< 0.001**		**0.004**		**< 0.001**
	No	14/50 (28%)		3/25 (12%)		11/25 (44%)	
	Yes	36/50 (72%)		22/25 (88%)		14/25 (56%)	
Post- education 2			**< 0.001**		**0.002**		**< 0.001**
	No	11/46 (24%)		1/23 (4%)		10/23 (43%)	
	Yes	35/46 (76%)		22/23 (96%)		13/23 (57%)	
Pre- education	**Examination by general practitioners**		Ref		Ref		Ref
	No	28/50 (56%)		5/25 (20%)		23/25 (93%)	
	Yes	22/50 (44%)		20/25 (80%)		2/25 (8%)	
Post- education 1			**< 0.001**		0.250		**< 0.001**
	No	13/50 (26%)		2/25 (8%)		11/25 (44%)	
	Yes	37/50 (74%)		23/25 (92%)		14/25 (56%)	
Post- education 2			**< 0.001**		0.219		**< 0.001**
	No	10/46 (22%)		1/23 (4%)		9/23 (39%)	
	Yes	36/46 (78%)		2/23 (96%)		14/23 (61%)	

*RTR* Renal transplant recipients

*GD* Glomerular diseases

## Discussion

Exposure to ultraviolet radiation is one of the primary modifiable risk factors of skin cancers. This study demonstrated an integrated skin cancer education program is effective in improving skin cancer awareness, regular self-skin examination and full skin examination by GPs in RTRs and patients with GD treated with long-term immunosuppressants. An increase in skin cancer and sun health knowledge after an integrated skin cancer education did not fully correlate to improved sun-protective practices.

Our recent survey highlighted the limited knowledge on skin cancer and inadequate photoprotective behaviours among RTRs and patients with GD in Central and Central West Queensland [29]. In our region, RTRs received formal skin cancer education prior to their transplant surgery. However, patients with GD did not receive formal skin cancer education prior to starting immunosuppressants. Although RTRs are expected to have higher skin cancer knowledge because of a prior education they received, there was no difference in mean SCSK score at baseline between the RTR cohort and GD group (Table 2). Our findings suggest that one-off education on skin cancer prior to transplantation is inadequate. There are likely multiple reasons for the latter including the timing of education and retention of knowledge during a challenging period for patients pre- and post-transplant surgery. In a small study with 25 transplant recipients, 80% preferred initial education to occur at ≥3 months post-transplant [11]. In another small study with 50 paediatric transplant patients, 85% of guardians and 73% of transplant recipients believed the best time to receive initial photoprotection and skin cancer education is before or immediately after transplantation [15]. Further studies are required to better evaluate when patients feel ready to receive initial skin cancer and photoprotection education.

Our findings are keeping with those of other studies among transplant recipients that skin cancer knowledge was improved when education was repeatedly administered [8–11, 13–16, 20]. There are currently no firm guidelines or consensus recommendations on the optimal timing to provide follow-up skin cancer education. Our study and others suggest that skin cancer knowledge increased in the immediate post-intervention period and is sustained at 3–8 months follow-up [13, 16]. Available data suggest skin cancer education should be reinforced at 6 monthly intervals.

Before skin cancer education, not all immunosuppressed renal patients in Central Queensland routinely performed skin self-examination or sought healthcare for skin checks despite having ultraviolet radiation and immunosuppressant-related skin cancer risks in these patients. Our findings support the provision of integrated skin cancer education after exposure to immunosuppressants is essential to enhance the rate of full skin examination by patients themselves and primary care medical practitioners.

Like this study, other studies also found that better skin cancer knowledge following the education did not always translate to an improvement in the utility and practice of various sun-protective methods [20, 22, 26]. Further research is required to understand barriers and facilitators of sun protective practices. In our study, there were higher sun protection rates in the RTR cohort which had a 72% of history of skin cancers which may facilitate sun protection practices. There are other factors, such as cultural, aesthetics, time consumed, costs, etc., that can influence photoprotective behaviours. The efficacy of education could be enhanced by incorporating videos and skin cancer images, content engagement that emphasise on behavioural and cultural aspects, as well as interactive materials and platforms [9, 10, 16]. Displaying skin protection posters, and availability of educational brochures, bookmarks and pamphlets on skin cancers and optimal sun-protection practices in clinic waiting rooms could supplement the efficacy of the education [8, 17]. In addition, the provision of dedicated staff in clinical settings may help educate and foster sun-protective behaviours in patients. Mobile devices such as tablet computers may facilitate education and training patients in a time-efficient, creative method [9]. Weather-dependent education or reminders via mobile medical apps may also positively influence sun-protective behaviours [8, 29]. Mihalis et al. proposed a model that included standard education (self-skin examination, using sun protection correctly, skin cancer education) plus a personalized behavioural plan, lesson follow up, uncovering misconception and summarising salient points to high risk patients [7]. A few studies reported that multimodal skin cancer education program is a promising strategy in improving skin cancer knowledge and sun protective behaviours [13, 21]. Health professionals need to establish better strategies to disseminate information and motivate patients in practicing effective sun protective behaviours. A collaboration among local skin organizations, transplant societies, and the International Transplant Skin Cancer Collaborative and Skin Care (https://www.itscc.org) is required to launch an integrated skin cancer program especially for transplant recipients who are high risk for invasive skin cancers and residing in countries with high UVI. This may lead to consensus guidelines in the optimal prevention and care of skin cancer in patients who are exposed to long-term immunosuppressant therapy.

Our study is subject to some limitations. As we focus on unique population in Central Queensland, a region receiving one of the highest dose or ultraviolet radiation in Australia, our sample size was relatively small and subject to potential sampling bias. All potential participants were approached and the first 25 patients who consented for each cohort were recruited into this pilot program. The interval of 3–6 months between education and administration of the study questionnaire was relatively short. Whilst the evaluation of long-term retention of skin cancer knowledge was not part of the study aim, it does warrant further consideration. In addition, self-reported skin cancer knowledge and sun-protective behaviours can be subject to recall bias. Strengths of our study include an excellent survey response rate with 94% completion at 6-months post-intervention. Participation was voluntary, and no incentives were provided to participants in this study. Approximately 40% of eligible patients in Central Queensland were included in the study and the data is likely to be representative of the Central Queensland Region. Generalizability can be applied to regional Caucasian RTRs and patients with GD in Australia and abroad. In a regional Australian setting, this is the first study that evaluated the effect of an integrated skin cancer education on skin cancer awareness and sun-protective practices among patients with two significant renal conditions requiring long-term immunosuppressants. The information included in the integrated skin cancer education program is robust and also based on literature from reputable cancer societies.

The comparison of skin cancer knowledge and photoprotective behaviours before and after skin cancer education between RTRs and patients with GD highlights the differences and similarities in relation to skin cancer education in individuals exposed to immunosuppressant therapy. As the follow-up period after skin cancer education is only 6 months, it is too early to determine the effect of educational intervention on incidence of skin cancer. An extended duration of follow-up study is being planned to better assess longer term aspects of skin cancer incidence before and after educational intervention in Central Queensland. This study will bring to light on whether integrated skin cancer education program results in lower incidence of skin cancer or less invasive disease.

## Conclusion

Single episode skin cancer education alone prior to immunosuppressive treatment is inadequate in RTRs. An integrated approach to skin cancer education should ideally improve awareness and reinforce sun-protective practice especially amongst patient populations who are more vulnerable. Further studies are required to establish means of improving compliance with sun-protective practices.

## Supplementary Information

**Additional file 1: Survey Questionary 1.** Skin Cancer and Sun Knowledge (SCSK) Scale Items. **Survey Questionary 2.** Outdoor activities, Sun Protection and Skin Examination.

## Data Availability

The datasets used and/or analysed during the current study will be available in a de-identifiable format from a corresponding author on reasonable request.
