# Supplementary material for: An integrated skin cancer education program in renal transplant recipients and patients with glomerular disease
Source: BMC Nephrol. 2022 Nov 10;23:361. doi: 10.1186/s12882-022-02997-z (PMC9647923; doi:10.1186/s12882-022-02997-z)
Supplement: Supplementary file 1 — Additional file 1: Survey Questionary 1. Skin Cancer and Sun Knowledge (SCSK) Scale Items. Survey Questionary 2. Outdoor activities, Sun Protection and Skin Examination. [file 12882_2022_2997_MOESM1_ESM.docx]

**Survey Questionary 1: Skin Cancer and Sun Knowledge (SCSK) Scale Items**

**Participant ID:**

**Date:**

**Survey Questionnaire 1**

1. I should stay out of the sun if my shadow is shorter than my body. **True** or False

2. Sunbathing for only a couple of weeks a year (e.g., when on holiday) increases your likelihood of getting skin cancer. **True** or False

3. Solariums/sun beds are a safe way to get a tan. True or **False**

4. When using sunscreen, you can tan without any negative effects. True or **False**

5. Having a tan protects my skin from the sun. True or **False**

6. A fake/spray on tan provides me with no protection from the sun. **True** or False

7. Keeping your skin tanned at a solarium during the winter protects it from sun damage during the summer. True or **False**

8. Gradual tanning eliminates most of the negative effects of lengthy exposure to the sun. True or **False**

9. A tan is a sign that the skin is damaged. **True** or False

10. UVR (ultraviolet rays) from tanning beds is safer than UVR from the sun. True or **False**

11. Tanning is an unsafe way to get the vitamin D your body needs. **True** or False

12. A tan is a sign of good health. True or **False**

13. If you are not usually exposed to the sun, being severely sunburned two or three times during your life will probably not increase your chances of skin disease. True or **False**

14. The only way a person can get skin cancer is from too much exposure to the sun. True or **False**

15. People with dark skin cannot get skin cancer. True or **False**

16. When should sunscreen be applied for best protection?

a. Just before you go in the sun

b. **15-30 minutes before going in the sun**

c. Within 15-30 minutes after going in the sun

17. How often should SPF 30 sunscreen be reapplied?

a. Every 30 minutes

b. **Every 2-3 hours and more often if swimming or sweating**

c. Neither of these options

18. When is the sun the strongest?

a. 9:00 a.m. to 12:00 p.m.

b. **10:00 a.m. to 4:00 p.m.**

c. 2:00 p.m. to 5:00 p.m.

19. Damage caused by the sun can be repaired by:

a. After-sun lotions (e.g., Aloe Vera lotion)

b. Moisturizers

c. Both of these options

d. **Neither of these options**

20. What type of clothing usually blocks more UV radiation (from the sun)?

a. Lighter colored clothing

b. **Darker colored clothing**

c. Both of these options

d. Neither of these options

21. What does SPF 30 mean?

a. **A sunscreen with a SPF of 30 means a person can stay in the sun 30 times longer without burning than he or she could if not wearing sunscreen**

b. A sunscreen with a SPF of 30 provides twice as much protection as a sunscreen with a SPF of 15

c. Both of these options

d. Neither of these options

22. Can you get a sunburn . . .

a. In a snowy environment?

b. In an outdoor pool or the ocean?

c. **Both of these places**

d. Neither of these places

23. Which of the following increases your risk of skin cancer?

a. Having had three severe sunburns in your past

b. Having a family history of skin cancer

c. **Both of these options**

d. Neither of these options

24. What is the most common form of skin cancer?

a. Melanoma

b. **Basal cell carcinoma (answer for non-transplant setting)**

c. **Squamous cell carcinoma (answer for transplant setting)**

d. None of these options

25. Which of the following could be a sign of skin cancer?

a. A sudden or gradual change in a mole’s appearance

b. A sore that doesn’t heal

c. **Both of these options**

d. Neither of these options

| Office use only: |
| --- |

**Survey Questionary 2: Outdoor activities, Sun Protection and Skin Examination**

**Participant ID: Date:**

**Survey Questionnaire 2**

1. How often do you do any outdoor activity (gardening, swimming, jogging, sports, etc.) for work or recreation or exercise under the sun? (Please circle)

- Almost daily/Daily
- Weekly
- Fortnightly
- Monthly
- Never
- Others (Please state) ……………………………………………………………

2. If you spend a lot of time outdoors, please circle as many statements relevant to you.

- Avoiding outdoor between 10am to 4pm
- Staying in the shade
- Wearing a hat
- Using an umbrella
- Wearing shirts with long sleeves
- Wearing light coloured clothes
- Wearing sunglasses
- Using sun protection cream (SPC)

1. How often do you use the cream? (Please circle)

- Never
- Rarely
- Sometimes
- Often/Always

1. Which season do you use sunscreen?

(Please circle as many statements as possible relevant to you)

- Summer
- Autumn
- Winter
- Spring

3. Do you perform self-skin examination regularly?

- No
- Yes (Specify frequency: …………… /6 months)

4. Do you have a full skin check regularly with your general practitioner?

- No
- Yes (Specify frequency: …………. /12 months)

**Self-skin examination**

**Who should do a self-skin examination?**

Everyone should! It is particularly important for people with the following characteristics to do so.

- White skin
- Aged over 40
- Previous [melanoma](https://dermnetnz.org/topics/melanoma/)
- Previous [basal cell carcinoma](https://dermnetnz.org/topics/basal-cell-carcinoma/) or [squamous cell carcinoma](https://dermnetnz.org/topics/cutaneous-squamous-cell-carcinoma/)
- Many [moles](https://dermnetnz.org/topics/mole/) or [freckles](https://dermnetnz.org/topics/brown-spots-and-freckles/)
- [Actinic keratoses](https://dermnetnz.org/topics/actinic-keratosis/) (scaly spots on sun-exposed sites)
- Outdoor work or recreation
- Many [sunburns](https://dermnetnz.org/topics/sunburn/) or visits to [solaria](https://dermnetnz.org/topics/sunbeds-and-solaria/)
- Syndromes that increase the risk of [skin cancer](https://dermnetnz.org/topics/skin-cancer/)
- Taking [immune suppressive medications](https://dermnetnz.org/topics/immunosuppressive-drugs/), e.g. organ transplant patients

## When should I examine my skin?

Skin self-examination should be done often enough to become a habit, but not so often as to feel like a bother. For most people an interval of one to three months is ideal.

## Steps of self-skin examination

- The self-skin examination should start with the head and end with the feet.
- The self-skin examination should include the scalp, face, neck, beneath facial hair and trunk.
- On the upper part of the body, the self-skin examination should include the armpits, hands, finger webs and nail beds.
- A mirror should be used to examine the back and skin between the buttocks.
- On the legs, it should include toe webs, toenail beds and soles of the feet.
- In your skin health diary, mark the site of any lesions you're concerned about.


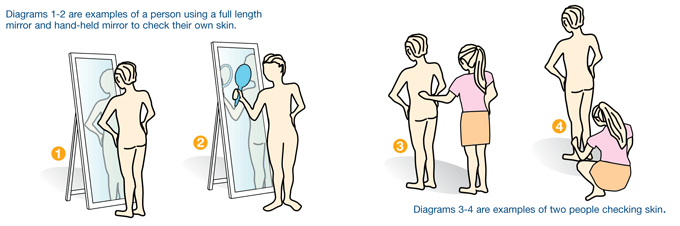


Image source: How to check your skin. Cancer Council (www.cancer.org.au)

Watch the video below for a step-by-step approach to a self-examination.

<https://www.sunsmart.com.au/skin-cancer/checking-for-skin-cancer>

**Best Practice Sun Protection Practices**

Image Source: World Health Organization (2002) Global UV Index: A practical guide

**Uniform, clothing and hat**

Wear a broad-brimmed, legionnaire or bucket hat that protects the face, neck, ears and crown of the head when outside.

Wear sun protective clothing (i.e. collared shirts, elbow or full-length sleeves, longer shorts, skirts or long pants).

Wear a rashie or similar top for swimming/water activities.

Wear appropriate sunglasses.

**Shade**

The use of shade (including temporary shade) is maximised during outdoor activities and indoor facilities are used wherever possible.

**Scheduling**

When outdoor activities are scheduled at times when the UV Index is 3 or higher, maximum use is made of shade, sunscreen, hats and long clothing to protect yourself.

**Sunscreen**

The use of SPF30 or higher broad spectrum, water resistant sunscreen is required before outdoor activities.

Time is given to apply sunscreen and reapplication after 2 hours is encouraged during extended outdoor periods.


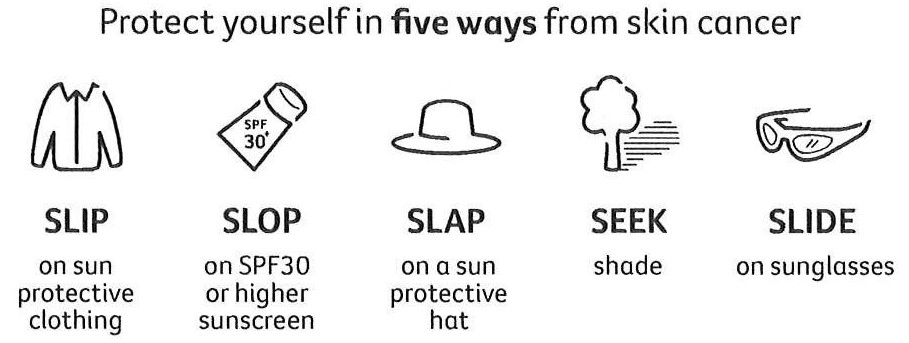


Image source: http://www.cancer.org.au/cancer-information/causes-and-prevention/sun-safety/be-sunsmart

**SKIN CANCER BROCHURE:**

**What is skin cancer?**

Skin cancers occur, when cells are damaged by ultraviolet (UV) light from the sun and use of solariums (sunbeds). Skin cancer can be found on any part of the body. Australia has the highest incidence of skin cancer in the world due to its high ultraviolet (UV) exposure rates resulting from our proximity to the equator.

**Why skin cancer education is needed?**

Studies show that transplant patients with high-level sun protection knowledge used sunscreen more effectively, and the skin cancer education they received not only improved their skin cancer awareness and photoprotective behaviours but also reduced their incidence of skin cancer.

**What are the types of skin cancer?**

**Non-melanoma skin cancers** (NMSC) are far more common than melanoma and are typically found on the head and neck in middle aged and older people due to long term sun exposure. People can also get skin cancers on their arms, legs and trunk. There are two common types of NMSC:

- **Basal cell carcinoma (BCC):** It usually grows slowly but can become very big and destroy tissue close to the tumour (locally), but rarely spread to other parts of the body (metastasise). It is the most common form skin cancer in the general population.
- **Squamous cell carcinoma (SCC):** It often starts as hard scaly skin (actinic keratosis) and abnormal cells (dysplasia) and SCC *in situ* (sometimes referred to as Bowen’s disease). A small number can travel to (metastasise) nearby lymph nodes, or be locally aggressive, and require more complex treatment. It is the most common form skin cancer in the kidney transplant recipients.

**Melanomas** are less common than NMSC and can occur in younger people often because of previous sunburns.

**Merkel cell carcinoma (MCC) (cancer of neuroendocrine cells in the skin)** is a rare skin cancer. It is most common in older people. Sun exposed areas, including the head and neck, arms and legs are the areas usually affected.

**What are the signs and symptoms of skin cancer?**

The signs and symptoms of skin cancer depend on where the cancer is, its size and how far it has spread in the body. There are several symptoms that may indicate skin cancer.

Common signs and symptoms may include:

- crusty, non-healing sores (or ulcers)
- small persistent lumps (these can be red, pale, or pearly)
- new spots, freckles, or lumps
- moles that change in thickness, colour, or shape over a period of weeks to months

**What causes skin cancer?**

Sun exposure is a major risk factor for skin cancer. The administration of immunosuppressive drugs further increases the risk of cancers.

Other factors that may increase the risk of skin cancer are

- Previous skin cancer
- Fair complexion and inability to tan (Please note that patient with dark skin can still get skin cancer)
- Poor immune system
- Family history of melanoma
- Moles on the skin
- Genetic predisposition

Image Source: World Health Organization (2002) Global UV Index: A practical guide

**What is UV Index?**

An ultraviolet (UV) index is a tool you can use to protect yourself from UV radiation. UV index reading of 1-2 means low danger from the Sun’s UV rays for the average person, 3-5 means moderate risk of harm from unprotected Sun exposure, 6-7 means high risk, 8-10 means very high risk and 11+ means extreme risk.

**When should I use the UV index?**

 Look or listen for the UV Index when you are:

- Planning or participating in an outdoor activity or event
- Undertaking recreational activities such as running, swimming, cycling or team sports
- Watching a spectator sport, such as tennis or cricket
- An outdoor worker, or have responsibility for outdoor workers, or
- Responsible for young children and their outdoor activities

**What is the shadow rule? (What your shadow can tell you about UV index?)**

The shadow rule is a simple way to know the peak time of day for UV exposure. If you shadow is taller than you, the UV exposure is lower and if you shadow is shorter than you, the UV exposure is greater.

Image source: <https://www.cancerresearchuk.org> ([https://t.co/HiJsKnnCic" / Twitter](https://t.co/HiJsKnnCic%22%20/%20Twitter))


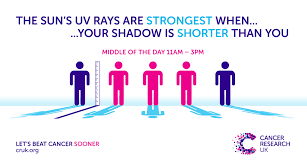


**Is Solarium safe?**

Image source: https://health.clevelandandclinic.org/tanning-bed-dangers/


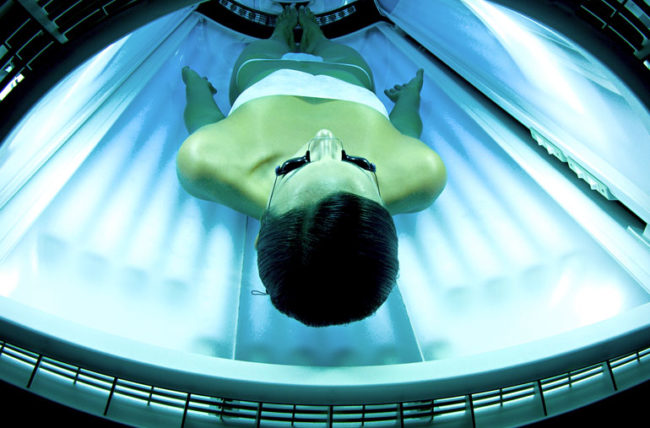


Solariums (Sunbeds) are machine that emit ultraviolet radiation and use of solariums increase the risk of skin cancer. There is no such thing as a safe tan- whether from a solarium or the sun. Tanning is a sign you skin cells are in trauma. Even if a tan fades, the damage remains. The more you tan your skin, the greater your risk of skin cancer. If you must have a tan, then use fake tan (spray) but remember that sun protection is still required.

**How do I protect my skin?**

One of modifiable risk factors in the prevention of skin cancer in renal transplant recipients is reducing patients’ exposure to ultraviolet exposure.

Image source: http://www.cancer.org.au/cancer-information/causes-and-prevention/sun-safety/be-sunsmart


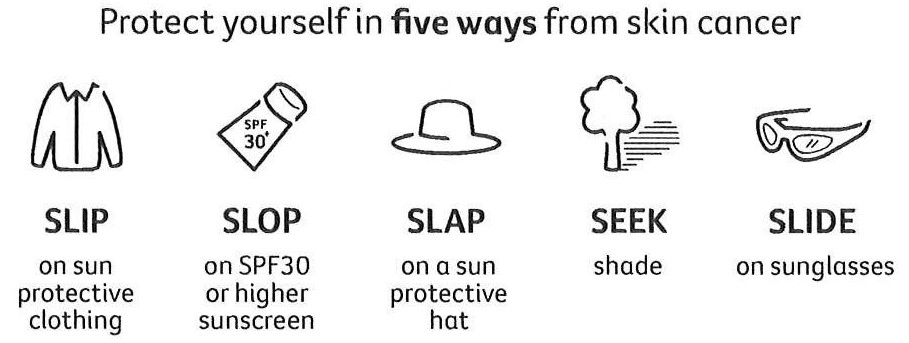


**What does SPF (sun protection factor) mean?**

The SPF number tells you how long the sun’s UV radiation would take to redden your skin when using the product exactly as directed versus the amount of time without any sunscreen. A sunscreen with a SPF of 30 means a person can stay in the sun 30 times longer without burning than he or she could if not wearing sunscreen.

Image source: https://www.skincancer.org


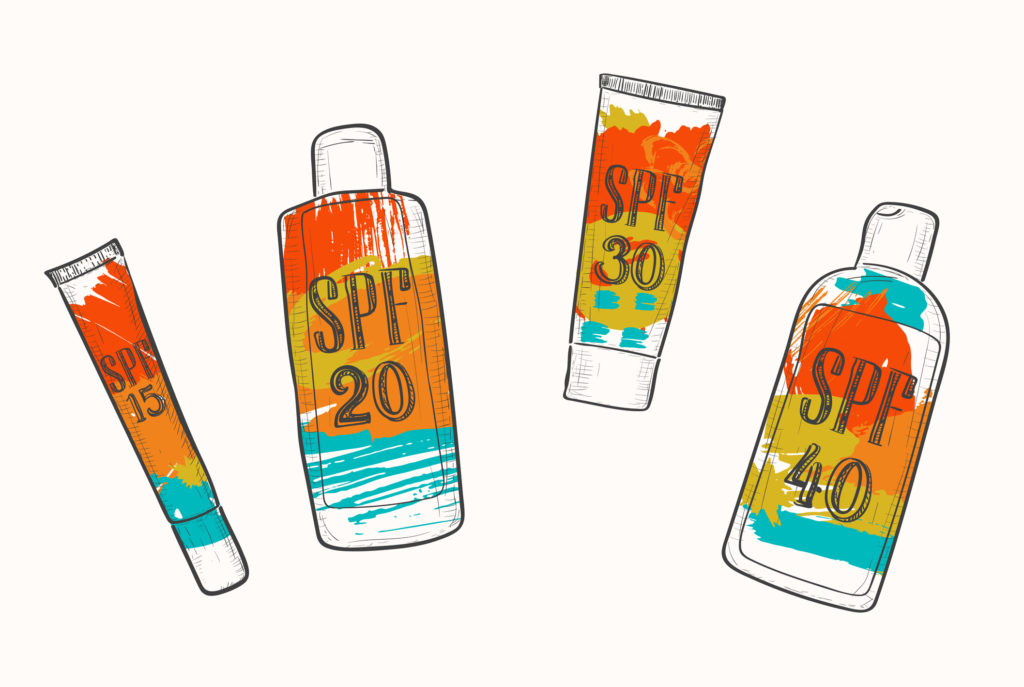


**Do you know the 5 W’S (& H) of Sunscreen?**

Image source: https://www.skincancer.org


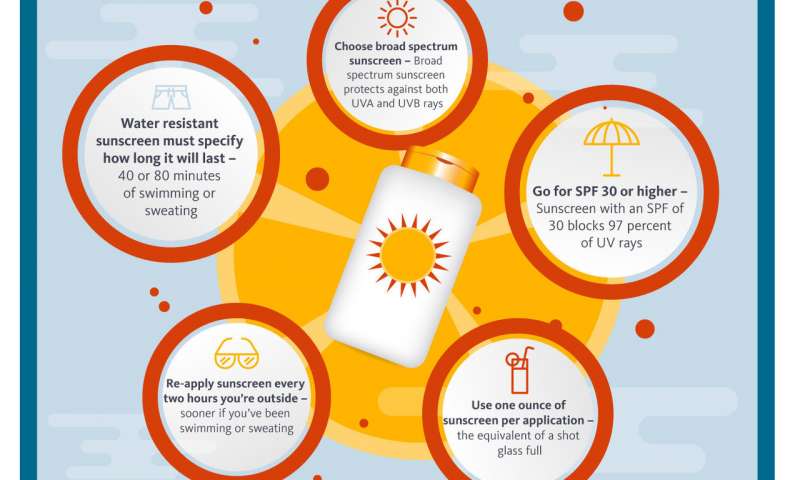


WHO: Everyone under the Sun

What: SPE30 or higher for a day outdoors

When: 30 minutes prior to going outdoors. Reapply every 2 hours.

Where: All exposed skin

How: One ounce (shot glass full) to entire body for each application

Why: Reduce your risk of skin damage and skin cancer

**Skin Health Diary**

Squamous cell cancer and basal cell cancer are the most prevalent skin cancers in renal transplant recipients and patients with glomerular disease treated with immune suppressive medications. Squamous cell cancer has significantly high mortality and morbidity in transplant recipients impacting the quality of life and overall prognosis. Cumulative incidence of post-renal transplant squamous cell carcinoma and basal cell cancer in Queensland was reported to be 52% after 10 years and 82% after 20 years post-transplantation. The incidence of squamous cell carcinoma and basal cell carcinoma in the Central Queensland region is likely higher due to greater ultraviolet sun exposure in the tropical climate.

Risk factors for skin cancers include ultraviolet sun exposure, previous history of skin cancer, fair skin complexion or phototype, age at transplantation, smoking, male sex, and viral infection with Human Papillomavirus. Prolonged use of immunosuppressive medications in organ transplant recipients enhance the existing risk factors.

We are keen to establish an integrated surveillance program with the assistance of patients, general practitioners and specialist team(s) which may aid the early diagnosis and management of skin cancers in renal transplant patients and patients with glomerular disease.

Skin cancer management is provided by many health care providers ranging from rural generalists, specialised skin clinics, general surgical and dermatology department at Rockhampton Hospital, general surgeons and plastic surgeons at a tertiary public hospital and private hospital. This diary allows patients to document finding of self-examination of skin and may improve communication among clinicians, which is important in the management of skin cancer.

**Preventing skin cancer**

Here are simple steps to reduce your skin cancer risk.

- Regular skin check and record information in this booklet so that your doctors can optimize level of immunosuppression to prevent rejection of kidney graft and developing infections and cancers
- Quit smoking
- Eat healthy diet
- Maintain a healthy weight
- Be SunSmart

Avoiding outdoor between 10am to 4pm if you can

Staying in the shade

Wearing a broad-brimmed hat

Using an umbrella

Wearing clothes with long sleeves

Wearing light coloured clothes

Wearing sunglasses

Using sun protection skin products (‘sunscreen’) (SPF 30 or higher)

| **General information: To be completed by treating kidney doctors and nurses.**  Height (cm):  Body weight (kg):  BMI:  Hair colour: Brown ם Blonde ם Black ם Red ם Auburn ם  Eyes colour: Blue ם Grey ם Brown ם Green ם Hazel ם Black ם  Skin type:   - always burns and never tans (pale white skin) (Type I) - always burns easily and tans minimally (white skin) (Type II) - burns moderately and tans uniformly (light brown skin) (Type III) - burns minimally and always tans well (moderately brown skin) (Type IV) - rarely burns and tans profusely (dark brown skin) (Type V) - never burns (deeply pigmented dark brown to black skin) (Type VI)   Skin cancer(s) prior to kidney transplantation: Yes ם No ם  Skin cancer(s) after kidney transplantation: Yes ם No ם  Previous kidney graft rejection: Yes ם No ם  Smoking: Current smoker ם Ex-smoker ם Non-smoker ם |
| --- |

**Date of skin Check:** **Performed by:**

Image: Body map according to the Systematized Nomenclature of Medicine (SNOMED) coding system. Buettner PG, Raasch BA. Int J Cancer. 1998; 78: 587-593.

| **Remark:**  **Name:**  **Date:**  **Position:** |
| --- |

Please ask your **general practitioner**, **skin doctors,** **kidney, or transplant doctors** to complete the following information whenever skin check or treatment is done.

Name: URN: DOB:

| **Site of a new skin lesion/ date noticed by a patient** | **Date of consultation for a new skin lesion** | **Type of skin procedure and date of the procedure** | **Is patient taking the following agents or procedures?** |
| --- | --- | --- | --- |
| Site:  **Date:**  / / |  | **Surgical excision** ם  **Cryosurgery** ם  **Electrodesiccation and curettage** ם  **Date:**  / / | **Acitretin Yes** ם No ם  **Nicotamide** Yes ם No ם  **Sirolimus/Everolimus** Yes ם No ם  **Capecitabine** Yes ם No ם  **Phototherapy** Yes ם No ם  **Radiation** Yes ם No ם  **Other Yes** ם No ם |
| **(Nephrologist only)**  Immunological risk for rejection: Low ם Intermediate ם High ם  Immunosuppressant level (Please specify the target): | | | |
| **Remark:**  **Date:**  **Name:**  **Position:** | | | |
